# The complete plastid genome of the endangered shrub *Brassaiopsis angustifolia* (Araliaceae): Comparative genetic and phylogenetic analysis

**DOI:** 10.1371/journal.pone.0269819

**Published:** 2022-06-30

**Authors:** Zhanghong Dong, Ruli Zhang, Ming Shi, Yu Song, Yaxuan Xin, Feng Li, Jianzhong Ma, Peiyao Xin

**Affiliations:** 1 Southwest Research Center for Landscape Architecture Engineering, National Forestry and Grassland Administration, Southwest Forestry University, Kunming, China; 2 Sympodial Bamboos Technological and Engineering Research Center, National Forestry and Grassland Administration, Southwest Forestry University, Kunming, China; 3 Key Laboratory of Ecology of Rare and Endangered Species and Environmental Protection (Ministry of Education), Guangxi Normal University, Guilin, China; 4 Yunnan Academy of Forestry and Grassland, Kunming, China; Institute for Biological Research, University of Belgrade, SERBIA

## Abstract

*Brassaiopsis angustifolia* K.M. Feng belongs to the family Araliaceae, and is an endangered shrub species in southwest China. Despite the importance of this species, the plastid genome has not been sequenced and analyzed. In this study, the complete plastid genome of *B*. *angustifolia* was sequenced, analyzed, and compared to the eight species in the Araliaceae family. Our study reveals that the complete plastid genome of *B*. *angustifolia* is 156,534 bp long, with an overall GC content of 37.9%. The chloroplast genome (cp) encodes 133 genes, including 88 protein-coding genes, 37 transfer RNA (tRNA) genes, and eight ribosomal RNA (rRNA) genes. All protein-coding genes consisted of 21,582 codons. Among the nine species of Araliaceae, simple sequence repeats (SSRs) and five large repeat sequences were identified with total numbers ranging from 37 to 46 and 66 to 78, respectively. Five highly divergent regions were successfully identified that could be used as potential genetic markers of *Brassaiopsis* and Asian Palmate group. Phylogenetic analysis of 47 plastomes, representing 19 genera of Araliaceae and two related families, was performed to reconstruct highly supported relationships for the Araliaceae, which highlight four well-supported clades of the *Hydrocotyle* group, Greater *Raukaua* group, *Aralia*-*Panax* group, and Asian Palmate group. The genus *Brassaiopsis* can be divided into four groups using internal transcribed spacer (ITS) data. The results indicate that plastome and ITS data can contribute to investigations of the taxonomy, and phylogeny of *B*. *angustifolia*. This study provides a theoretical basis for species identification and future biological research on resources of the genus *Brassaiopsis*.

## Introduction

The genus *Brassaiopsis* Decne. & Planch. in the family Araliaceae includes nearly 45 species, with most of the species in the Himalayas, western China, Indochina, and the Malay Peninsula [1, 2], with southwestern China and northern Indochina as the main center of diversity [1]. The genus is part of the core Asian clade of Araliaceae [1, 3–5], and the species delimitation and infrageneric classification of *Brassaiopsis* has been highly controversial [1]. *Brassaiopsis angustifolia* K.M. Feng is an endangered shrub species. There is an urgent need to manage and conserve the natural resource of this shrub. However, information on its genetic and genomic background is limited. At present, the systematic position of *B*. *angustifolia* is not clear in the genus of *Brassaiopsis*. Therefore, knowledge of the genomics and phylogeny of its populations is essential to formulating effective protective measures.

During the past 20 years, molecular data have greatly improved our understanding of the phylogenetic relationships within the Araliaceae family and within the Apiales order [6]. Previous phylogenetic studies based on nuclear ribosomal DNA [1–5, 7–12] and chloroplast (cp) DNA [5, 7–13] sequence data have provided important clues about the evolution and diversity of Araliaceae plants. However, traditional phylogenies based on analysis of multiple genes have failed to well solve the relationship among species of Araliaceae. In addition, the early divergences of the Asian Palmate group have been clarified by using the chloroplast genome, but the backbone of its core is not totally resolved [14]. The family of Araliaceae, divided into four main clades by molecular systematics: Greater *Rauakaua*, *Polyscias*–*Pseudopanax*, *Aralia*–*Panax*, and Asian Palmate, respectively [3, 5, 10, 14]. Although several recent studies have made progress, sampling from Araliaceae has remained limited largely in most studies because of a focus on problems at other phylogenetic levels, individual geographic regions, or questions dedicated to a single genus [1, 2, 7, 8, 12]. The genus *Brassaiopsis* is part of the core Asian clade of Araliaceae. Based on the comprehensive analysis of internal transcribed spacer (ITS) and cp DNA data, the sister group relationship of *Brassaiopsis* and *Trevesia* were supported [1–3, 5, 7–11]. Because of low sequence quality, the few samples, and rapid divergence in early evolutionary history, the phylogenetic relationship among genera and species has not yet been well solved of Araliaceae [2–5, 9, 11, 15]. In addition, the species delimitation and infrageneric classification of *Brassaiopsis* are highly controversial. Therefore, it is necessary to use the existing cp genome data to construct a robust phylogenetic tree to clarify the phylogenetic relationships within Araliaceae.

In plants, the cp is the main locus of photosynthesis and carbon fixation [16, 17]. The cp genome of higher plants is a double-stranded circular DNA molecule ranging in size from 72 to 217 kb, containing about 130 genes. The cp genome has a typical tetrad structure, including a large single copy (LSC) region, a small single copy (SSC) region, and a pair of inverted repeats (IRs) in most plants [17–19]. Compared with nuclear genomes, the uniqueness of the cp is evident in its maternal inheritance, small size, simple structure, and conserved sequences [20, 21]. The cp genome sequence reveals the phylogenetic relatedness visually at different taxonomic levels and provides an understanding of the evolution of a plant’s structure and function [22, 23]. Therefore, the genome sequence is widely used for cp inheritance, domestication studies, phylogeny, and adaptive evolution.

Here, we report for the first time, to our knowledge, the complete chloroplast genome sequence of *B*. *angustifolia* and characterize the structure, gene content, and organization of its genome. Then, we establish its codon usage frequencies, simple sequence repeats (SSRs), repeats, regions of high sequence divergence, nucleotide variability values, and the expansion and contraction of its IRs. Finally, we evaluated the phylogenetic position by comparative analysis based on 42 entire plastid genomes sequences of Araliaceae species and conducted a phylogenetic analysis of the *Brassaiopsis* genus using nuclear ribosomal ITS data. The results of this study can provide clues for species classification of *Brassaiopsis* and help to clarify the evolution and phylogenetic relationships of the species and genera of the Araliaceae family. Furthermore, this newly developed genomic resource will help further conserve the genetics of this endangered species.

## Materials and methods

### Sampling, DNA extraction and sequencing

We collected fresh and young leaves of *B*. *angustifolia* from a cultivated tree at the Southwest Forestry University, Kunming, China (102°45.489′ E, 25°3.639′ N). Total genomic DNA was extracted using the modified cetyltrimethylammonium bromide (CTAB) method [24]. Long-range polymerase chain reaction was performed following Zhang et al. [25], with 15 pairs of universal primers. The entire cp genome of *B*. *angustifolia* was sequenced via 250 bp paired-end sequencing on a HiSeq 2500 Platform (Illumina, Nanjing, China).

### Analysis of cp genome assembly, annotation, and relative synonymous codon usage

Raw reads were filtered to remove low-quality reads, and de novo assembly of circular plastome and ITS sequence were carried out using GetOrganelle software [26]. We used Bandage software [27] to examine and screen the assembled cp genome of *B*. *angustifolia*. The cp genome was adjusted and annotated with Geneious software [28], and use the Organelle Genome DRAW software [29] to drawn a circular structure diagram of the entire genome. The entire annotated chloroplast genome and the ITS sequences were submitted to the National Center for Biotechnology Information (cp genome GenBank accession: OK638200; ITS GenBank accession: OL352055). Relative synonymous codon usage (RSCU) and codon usage were examined with CodonW (version 1.4.4) [30].

### SSRs and identification of repeats

Using the MicroSatellite (MISA) identification tool [31], the minimum repeat number of mononucleotides was 10; for dinucleotides, it was five, and for trinucleotide, tetranucleotide, pentanucleotide, and hexanucleotide repeat motifs, it was four. The SSRs of 1–10 units of the nine complete cp genomes of the Asian Palmate group were detected. Complement repeats, forward repeats, palindromic repeats, and reverse repeats in non-SSR of the nine cp genomes were detected with the online software REPuter (https://bibiserv.cebitec.uni-bielefeld.de/reputer) [32], using the parameter default values. Tandem repeats in non-SSR of nine cp genomes were detected with online software TRF (http://tandem.bu.edu/trf/trf.basic.submit.html) [33].

### Comparative genome analysis and sequence divergence

MAFFT software [34] was used to align the nine cp genomes of the Asian Palmate group, which were then manually adjusted with BioEdit software [35]. Sequence identity analysis was visualized with the mVISTA program in Shuffle-LAGAN mode [36], with the annotation for *B*. *angustifolia* as a reference. We performed a sliding-window analysis in the DnaSP software (version 6) [37] to evaluate the variability(π) of the plastomes. The window length was set to 600 bp and the step size to 200 bp. The contraction and expansion of the IR boundaries of nine species in the Asian Palmate group were visualized with the IRscope software [38].

### Phylogenetic analysis based on cp genome and ITS data

Two different data matrices were assembled and analyzed using both maximum likelihood and Bayesian inference (BI) methods. Matrix I contained 42 taxa with complete plastid genomes available (S1 Table), including members of *Aralia* L. (5 spp.), *Brassaiopsis* Decne. & Planch. (2 spp.), *Cheirodendron* Nutt. ex Seem. (1 sp.), *Chengiopanax* C. B. Shang & J. Y. Huang (1 sp.), *Dendropanax* Decne. & Planch. (4 spp.), *Eleutherococcus* Maxim. (5 spp.), *Fatsia* Decne. & Planch. (2 spp.), *Hedera* L. (2 spp.), *Heptapleurum* Gaertn. (3 spp.), *Heteropanax* Seem. (1 sp.), *Hydrocotyle* L. (1 sp.), *Kalopanax* Miq. (1 sp.), *Macropanax* Miq. (1 sp.), *Merrilliopanax* H. L. Li (1 sp.), *Oplopanax* Miq. (1 sp.), *Panax* L. (6 spp.), *Raukaua* Seem. (3 spp.), *Schefflera* J. R. Forst. & G. Forst. (1 sp.), and *Tetrapanax* (K. Koch) K. Koch (1 sp.) (S1 Table). *Angelica keiskei* (Miq.) Koidz. (GenBank accession: MW125613), *Diplopanax stachyanthus* Hand.-Mazz. (GenBank accession: MG524991), *Diplopanax stachyanthus* Hand.-Mazz. (GenBank accession: KP318983), and *Ostericum grosseserratum* (Maxim.) Kitag. (GenBank accession: KT852844), were sampled as outgroups. Matrix II included 24 species of *Brassaiopsis* and eight species of *Trevesia*, and included 34 ITS sequences (S2 Table).

The complete cp genome and ITS matrix were aligned with MAFFT software (version 7) [34] and then manually edited with BioEdit 7.2.5 [35]. Bayesian inference was undertaken using MrBayes (version 3.2.6) [39]. Used jModelTest (version 2.1.10) [40] to select the most suitable replacement DNA model for phylogenetic reconstruction. The Markov chain Monte Carlo (MCMC) algorithm was run for 10,000,000 generations. The first 25% of the trees was discarded as aging trees and the remaining trees were used to generate a consensus tree for majority-rule. When the average standard deviation of split frequencies was less than 0.01, we considered the operation to be stable. Nodes with posterior probability (PP) values of 0.95 or greater were considered statistically significant. ML analysis was performed IQ-TREE (version 1.6.7) [41]. ML bootstrap support (BS) values of 70% or greater were considered well supported, and ML BS of less than 50% were considered poorly supported or unresolved. The best‐fit DNA substitution models for matrix I and matrix II, respectively, were chosen as “TPM1uf + I + G” (freqA = 0.3138, freqC = 0.1857, freqG = 0.1807, freqT = 0.3197, R (a) [AC] = 1.0000, R(b) [AG] = 1.6623, R(c) [AT] = 0.4054, R (d) [CG] = 0.4054, R(e) [CT] = 1.6623, R(f) [GT] = 1.0000, p-inv = 0.5260, and gamma shape = 0.9030) and “TrNef + G” (R(a) [AC] = 1.0000, R(b) [AG] = 4.0619, R (c) [AT] = 1.0000, R(d) [CG] = 1.0000, R(e) [CT] = 7.9724, R(f) [GT] = 1.0000, and gamma shape = 0.1830) to construct the phylogenetic tree. Results of all phylogenetic analysis are displayed with FigTree (version 1.4.3) (http://tree.bio.ed.ac.uk/publications/).

## Results

### Chloroplast genome characteristics of *B*. *angustifolia*

**Fig 1:**
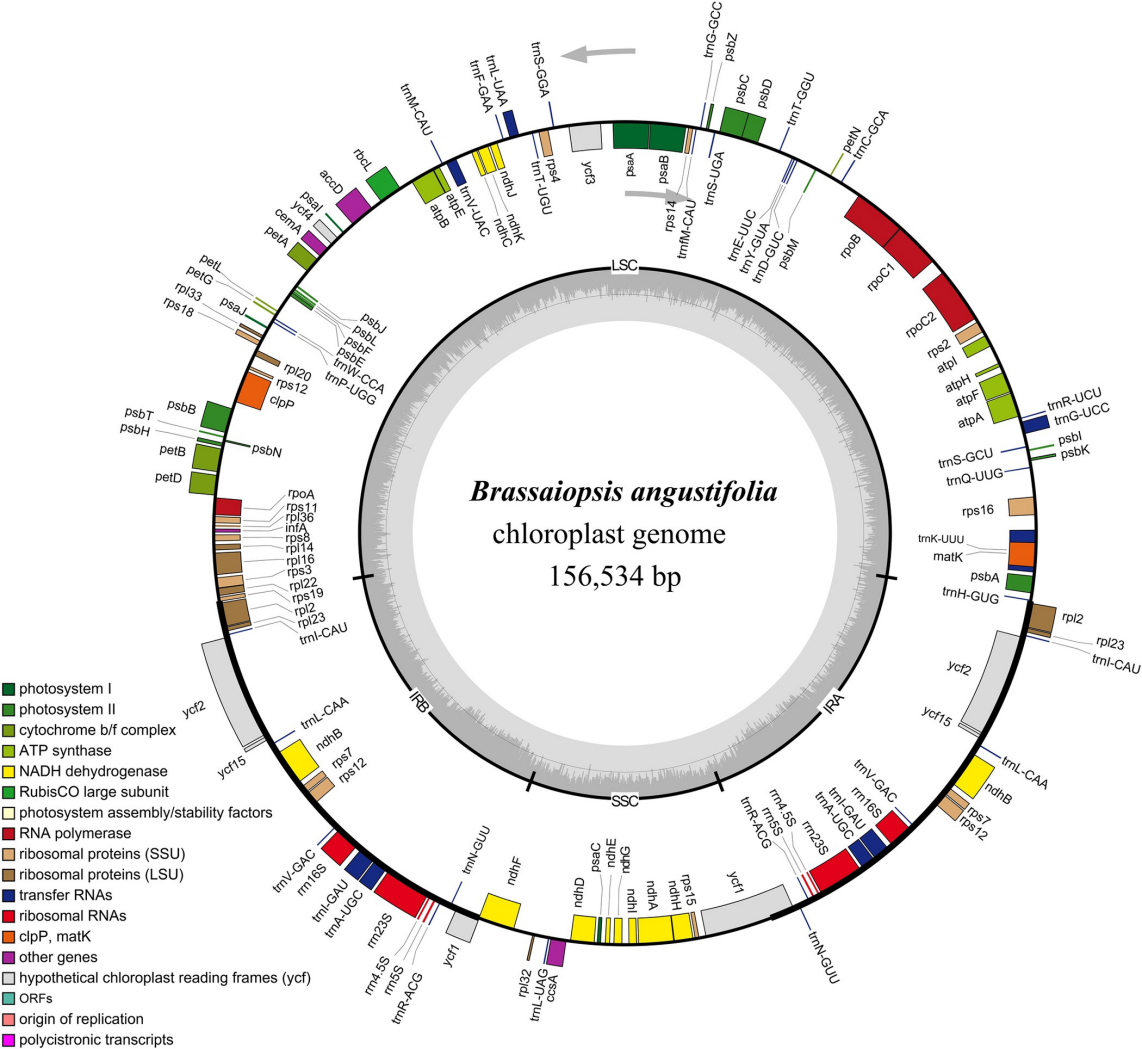
Circular map of chloroplast genome of *Brassaiopsis angustifolia* with annotated genes. Genes shown inside and outside of the circle are transcribed in clockwise and counterclockwise directions, respectively. Genes belonging to different functional groups are color‐coded. The GC and AT content are denoted by the dark gray and light gray colors in the inner circle, respectively.

The size of the *B*. *angustifolia* cp genome was 156,534 bp, and exhibited the usual quadripartite structure, featuring a LSC region (86,509 bp), a SSC region (18,061 bp), and a pair of IRs (25,982 bp) (Fig 1). The guanine-cytosine (GC) content was 37.9% in whole cp, 36.1% and 31.9% in the LSC and SSC regions, respectively, whereas higher rates (43.0%) were distributed in the IR regions. A total of 133 genes were annotated in the sequenced *B*. *angustifolia* cp genome, containing 88 protein-coding genes, 37 transfer RNAs (tRNAs), and eight ribosomal RNAs (rRNAs) (S3 Table). These genes belong to several categories with different functions; among which 17 duplicated genes were located in the IR regions, including six protein-coding genes (*ndhB*, *rpl2*, *rpl23*, *rps7*, *ycf2*, and *ycf15*), seven tRNAs (*trnA*-*UGC*, *trnI*-*CAU*, *trnI*-*GAU*, *trnL*-*CAA*, *trnN*-*GUU*, *trnR*-*ACG*, and *trnV*-*GAC*), and four rRNAs (*rrn4*.*5S*, *rrn5S*, *rrn16S*, and *rrn23S*). The transcription regulation of genes was believed to be affected by introns and exons. Nineteen genes (11 protein-coding genes and eight tRNAs) contained at least one intron, and three genes (*clpP*, *ycf3*, *and rps12*) had two introns (S3 Table).

**Fig 2:**
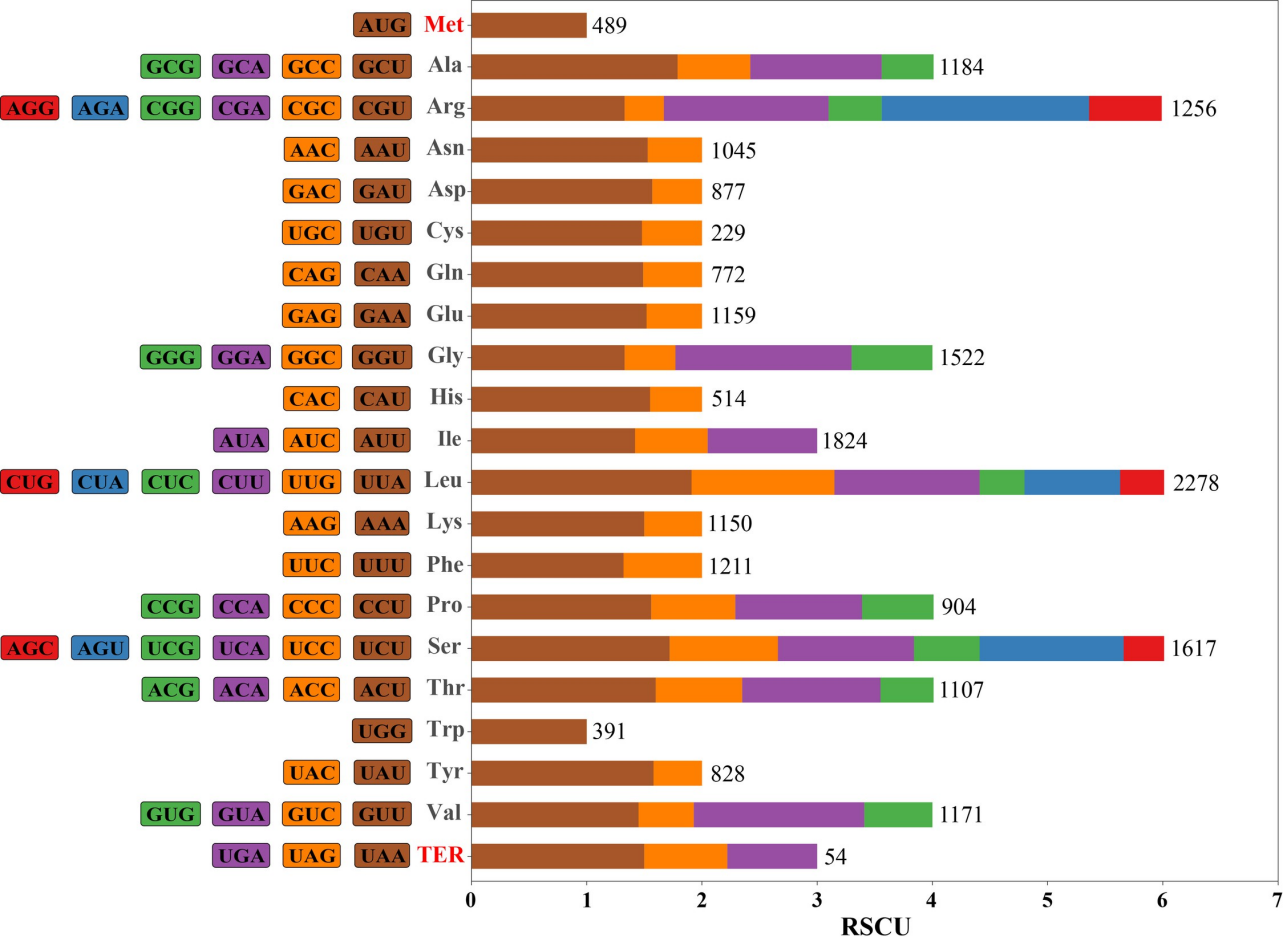
Relative synonymous codon usage (RSCU) analysis of 20 amino acids and three termination codons in *Brassaiopsis angustifolia*. Bar diagram in different colors reflects codon usage bias (brown, yellow, purple, green, blue, and red, correspond to the proportion of different codons in descending order). Numbers represent codon quantities.

According to the RSCU analysis (Fig 2), all protein-coding genes consisted of 21,582 codons. Among them, leucine (2,278 codons, 10.56%) was the most abundant amino acid, and isoleucine (1,824 codons, 8.45%) was the second. Each amino acid corresponded to at least one codon (tryptophan) and up to six (arginine, leucine, methionine, and serine). Trp had only one codon (UGG), which meant there was no codon usage bias. In addition, with the exception of methionine, most amino acids tended to use codons that ended in A/U rather than in C/G.

### Analysis of SSR and repeats for the nine cp genomes of the Asian Palmate group

**Fig 3:**
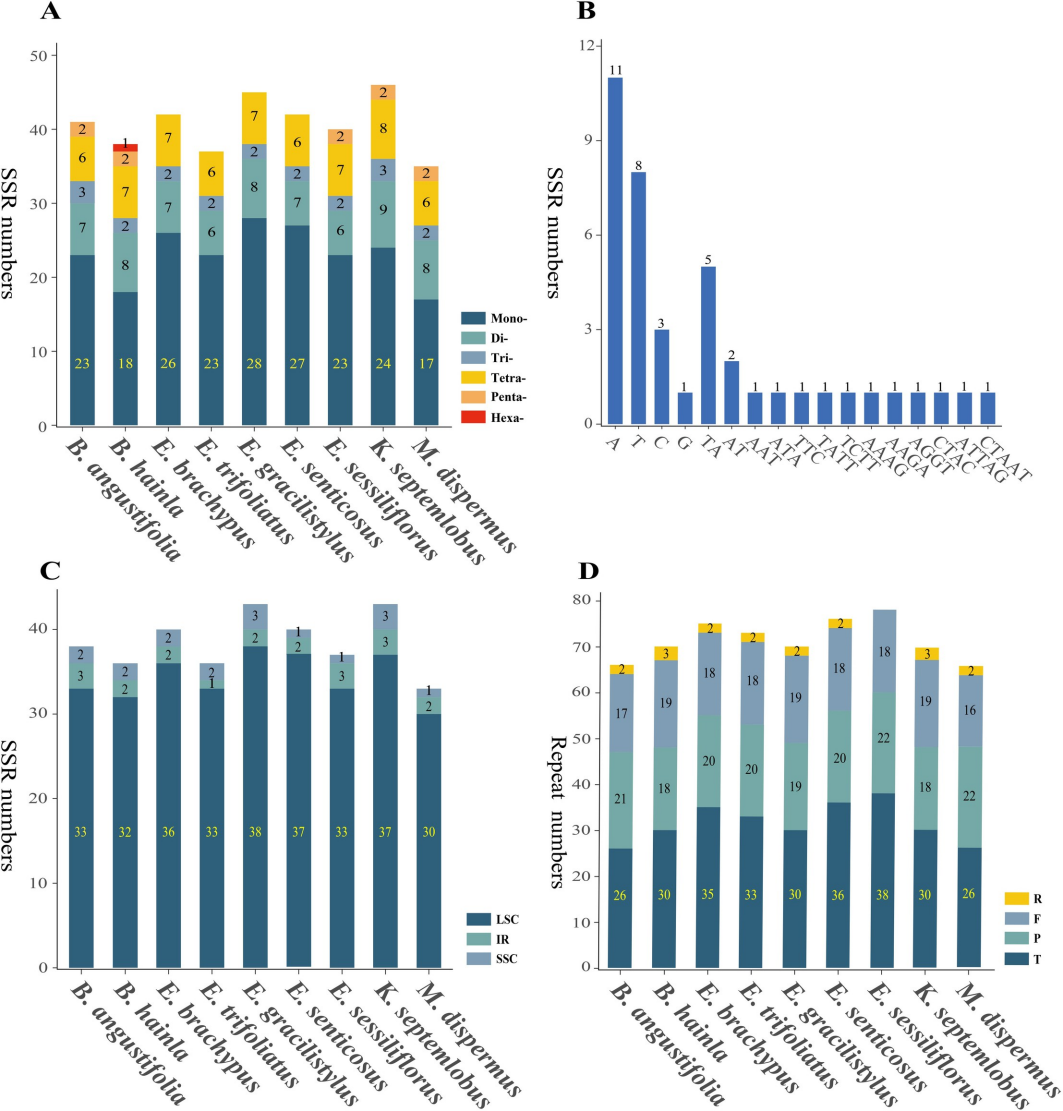
The simple sequence repeat (SSR) elements in the chloroplast genome of nine species in the Asian Palmate group. (A) Number of six SSR types. (B) Number of identified SSR motifs of *Brassaiopsis angustifolia*. (C) Frequency of identified SSRs in large single copy (LSC) regions, small single copy (SSC) regions, and inverted repeat (IR) regions. (D) Total of four repeat types.

MISA analysis of 9 species cp genomic sequences from the Asian Palmate group revealed numerous SSR loci. In total, six types of SSRs (mononucleotide, dinucleotide, trinucleotide, tetranucleotide, pentanucleotide, and hexanucleotide repeats) were detected based on the comparison of nine genomes in the Asian Palmate group. A total of 41 perfect SSRs were found in *B*. *angustifolia* (Fig 3A). Similarly, 38, 42, 37, 45, 42, 40, 46, and 35 SSRs were detected in *Brassaiopsis hainla* (Buch.-Ham.) Seem., *Eleutherococcus brachypus* Nakai, *Eleutherococcus trifoliatus* (L.) S. Y. Hu, *Eleutherococcus gracilistylus* (W. W. Sm.) S. Y. Hu, *Eleutherococcus senticosus* Maxim., *Eleutherococcus sessiliflorus* (Rupr. & Maxim.) S. Y. Hu, *Kalopanax septemlobus* Koidz., and *Macropanax dispermus* Kuntze. The most abundant type of SSR was mononucleotide repeats, which ranged from 17 bp in *M*. *dispermus* to 28 bp in *E*. *gracilistylus*, followed by dinucleotide, tetranucleotide, trinucleotide, pentanucleotide, and hexanucleotide repeats (Fig 3A). In the cp genome of *B*. *angustifolia*, most mononucleotide SSRs had A (47.83%) and T (34.78%) motifs, whereas all dinucleotide repeats were composed of TA (74.43%) and AT (28.57%) motifs (Fig 3B). Further analysis shows that most of the microsatellites were in the LSC region and a small proportion in the SSC and IR regions (Fig 3C). A total of 66 repeats were found in the *B*. *angustifolia* chloroplast genome, including tandem, palindromic, forward, inverted, and complement repeats. Similarly, 70, 75, 73, 70, 76, 78, 70, and 66 repeats were detected in *B*. *hainla*, *E*. *brachypus*, *E*. *trifoliatus*, *E*. *gracilistylus*, *E*. *senticosus*, *E*. *sessiliflorus*, *K*. *septemlobus*, and *M*. *dispermus*, respectively (Fig 3D).

### Identification of mutational hotspots among nine species of the Asian Palmate group

**Fig 4:**
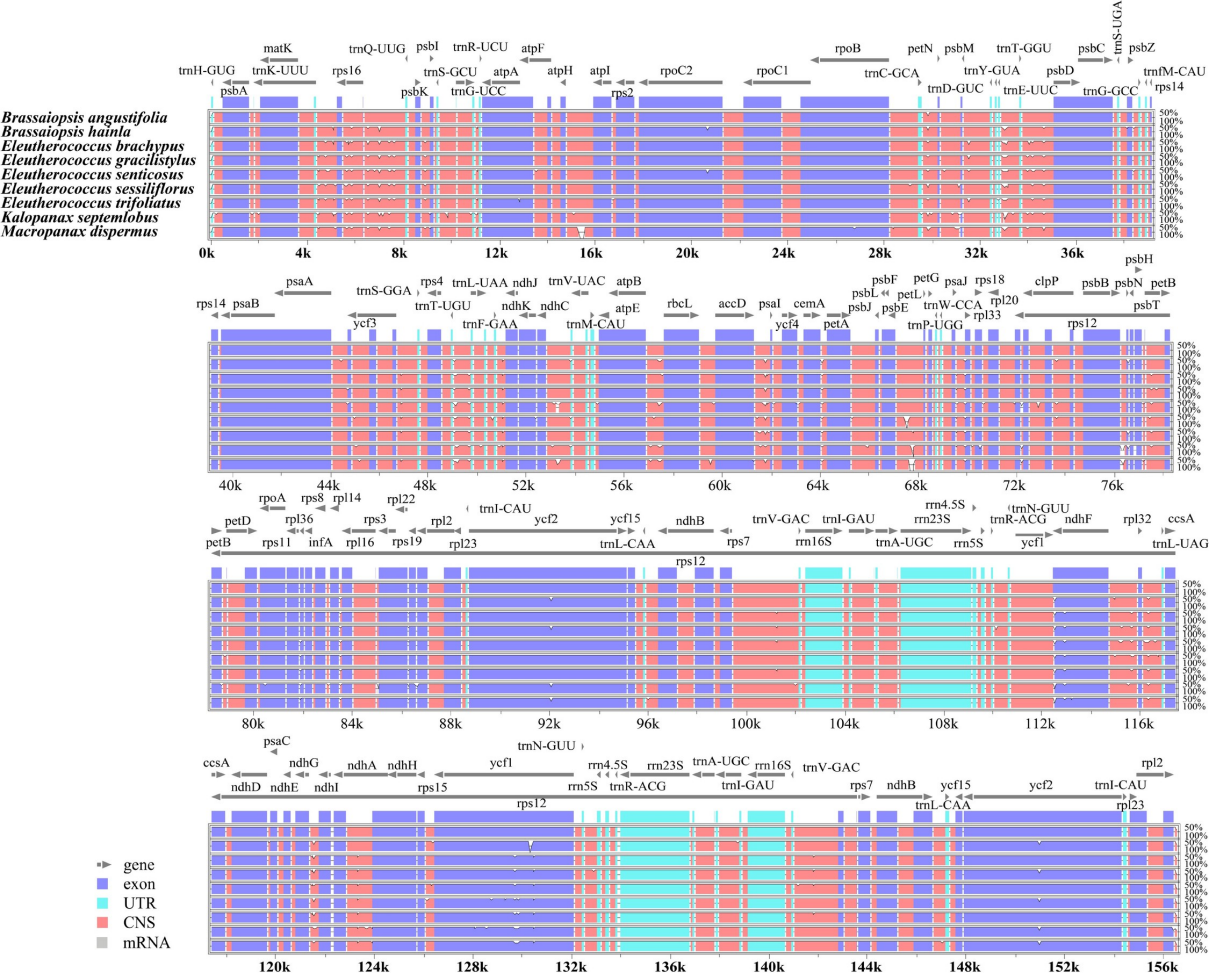
Complete chloroplast genome alignments for nine species of the Asian Palmate group using the mVISTA program, with the *Brassaiopsis angustifolia* chloroplast genome as a reference sequence. The y-scale indicates identity from 50% to 100%. Gray arrows indicate the position and direction of each gene. Red indicates conserved non-coding sequences (CNSs). Blue indicates the exons of protein-coding genes (exon).

Sequence identity analysis was performed using mVISTA (Fig 4). Multiple sequence alignment revealed high similarity among the nine chloroplast genomes of the Asian Palmate group, which suggests that they are highly conserved; specifically, the results showed that the divergence of the single-copy (SC) region was larger than that of the IR region, and the divergence of the non-coding region was larger than that of the coding region.

**Fig 5:**
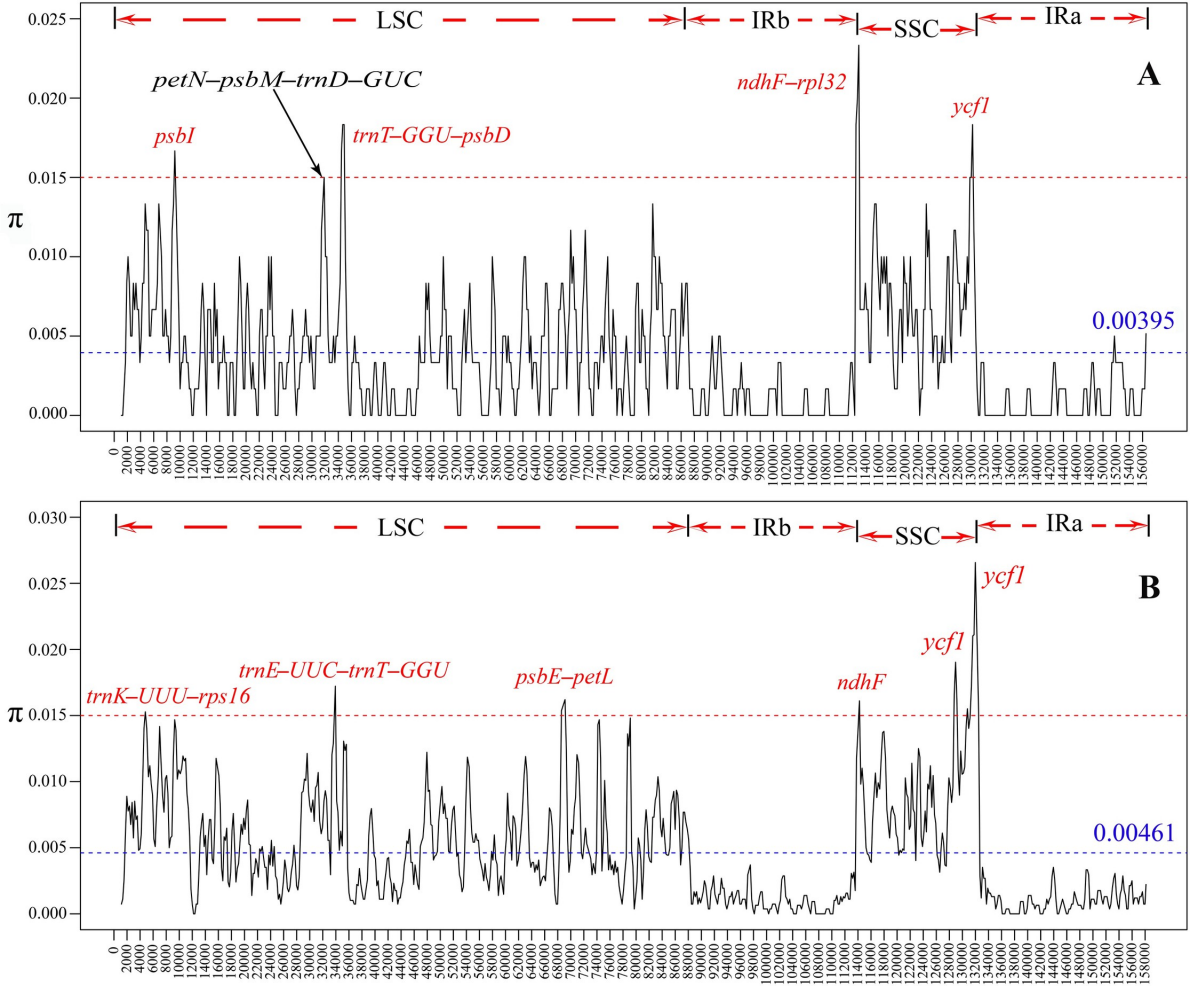
Comparison of the nucleotide variability (π) values of the two *Brassaiopsis* plastomes (A) and nine plastomes of the Asian Palmate group (B). (Window length, 600 bp, step size, 200 bp); x-axis: position of the midpoint of a window; y-axis: nucleotide diversity of each window.

The software DnaSP 6.0 was used to calculate the nucleotide variation value (π) within 600 bp of the cp genome of *B*. *angustifolia*, *B*. *hainla*, *E*. *brachypus*, *E*. *gracilistylus*, *E*. *senticosus*, *E*. *sessiliflorus*, *E*. *trifoliatus*, *K*. *septemlobus*, and *M*. *dispermus*. The difference between the two *Brassaiopsis* species the value varied from 0 to 0.02333, with an average of 0.00395, suggesting that their genomic differences were small. However, five highly variable loci with much higher π values (π > 0.015), including the *psbⅠ*, *petN*-*psbM*-*trnD*-*GUC*, *trnT*-*GUU*-*psbD*, *ndhF*-*rpl32*, and *ycf1*, which were precisely located (Fig 5A). Among seven species of the Asian Palmate group and the two *Brassaiopsis* species, the π values varied from 0 to 0.02657 with a mean of 0.00461, indicating that the differences among species of the Asian Palmate group were larger than those between congeneric species. Five highly variable loci including the *trnK*-*UUU*-*rps16*, *trnE*-*UUC*-*trnT*-*GGU*, *psbE*-*petL*, *ndhF*, and *ycf1* were precisely located in the nine species of the Asian Palmate group (π > 0.015; Fig 5B).

### Expansion and contraction of the inverted repeat regions

**Fig 6:**
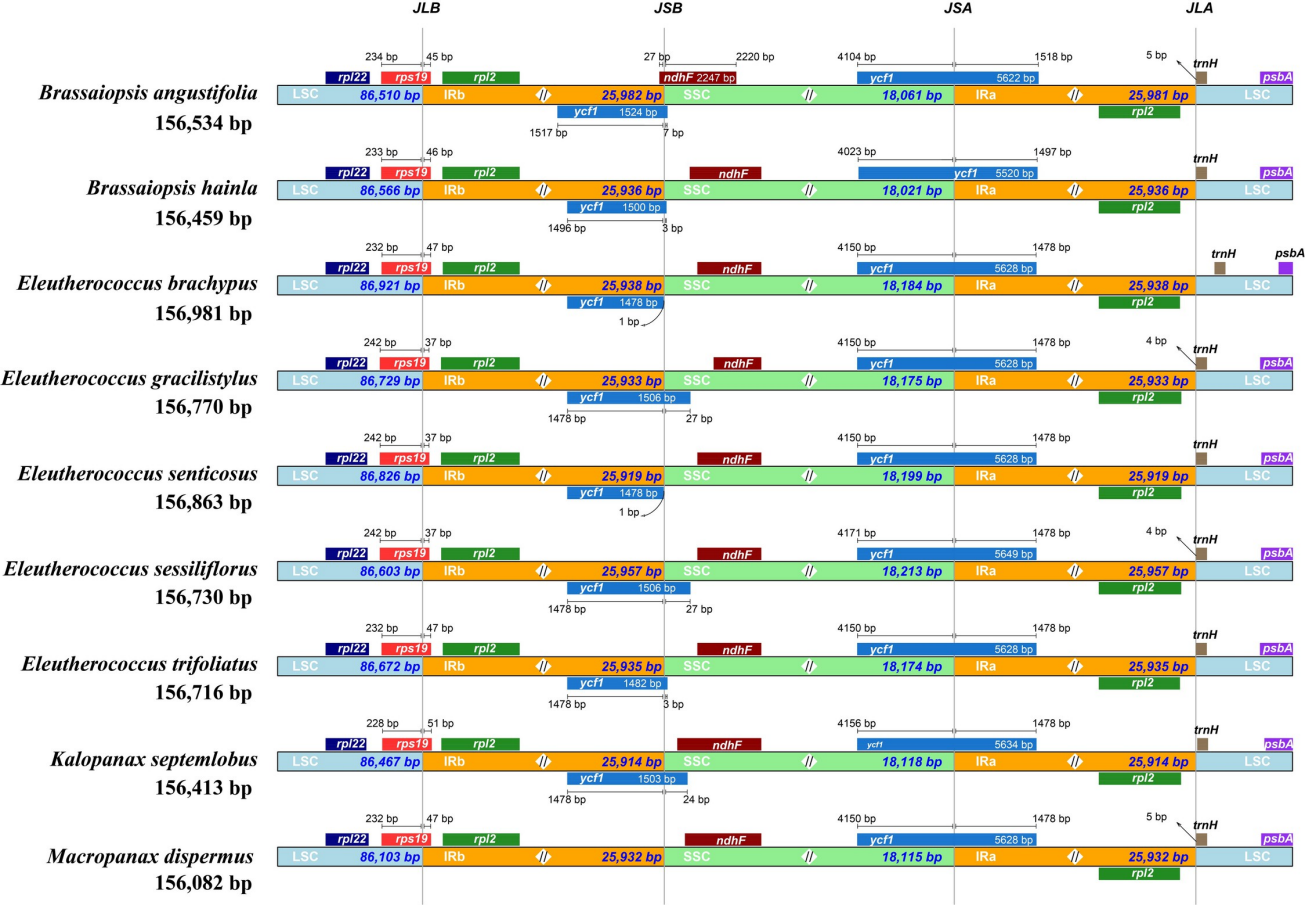
Comparison of the borders of the inverted repeat (IR), small single copy (SSC), and large single copy (LSC) regions among nine chloroplast genomes of the Asian Palmate group. JLB, JSB, JSA, and JLA represent the junctions of LSC/IRb, IRb/SSC, SSC/IRa, and IRa/LSC, respectively. The Fig is not drawn to scale based on sequence length but shows only the relative change near or at the IR/SC junctions.

The IR boundaries of *B*. *angustifolia* and eight species of the Asian Palmate group were compared, and the possible expansion or contraction of IR regions was analyzed (Fig 6). The expansion and contraction of the boundaries of chloroplast genomic IR regions in nine plants of the Asian Palmate group were revealed, and the four junctions of two IRs between *B*. *angustifolia* and its related species were compared in detail (Fig 6). By comparing the plastids of the Asian Palmate group species, we found that the IR/LSC connections of IRb were mainly located between *rpl2* and *rps19* genes (Fig 6). In addition, the overlap of *ycf1* genes appeared in different positions among the Asian Palmate group species, that is, the SSC region of *B*. *angustifolia* and the IRB/SSC junction of other seven species. *M*. *dispermus* did not contain the *ycf1* gene. The *ycf1* genes sited at the SSC/IRa boundary and the length of *ycf1* ranged from 5,520 to 5,649 bp. The *trnH* genes of the four species in the Asian Palmate group were located in the LSC region, 2–5 bp away from the IRa-LSC border. In *B*. *angustifolia*, the expansion of the LSC region leads to the *ndhF* gene being at the IRb/LSC junction.

### Phylogenetic relationships among the Araliaceae genera

**Fig 7:**
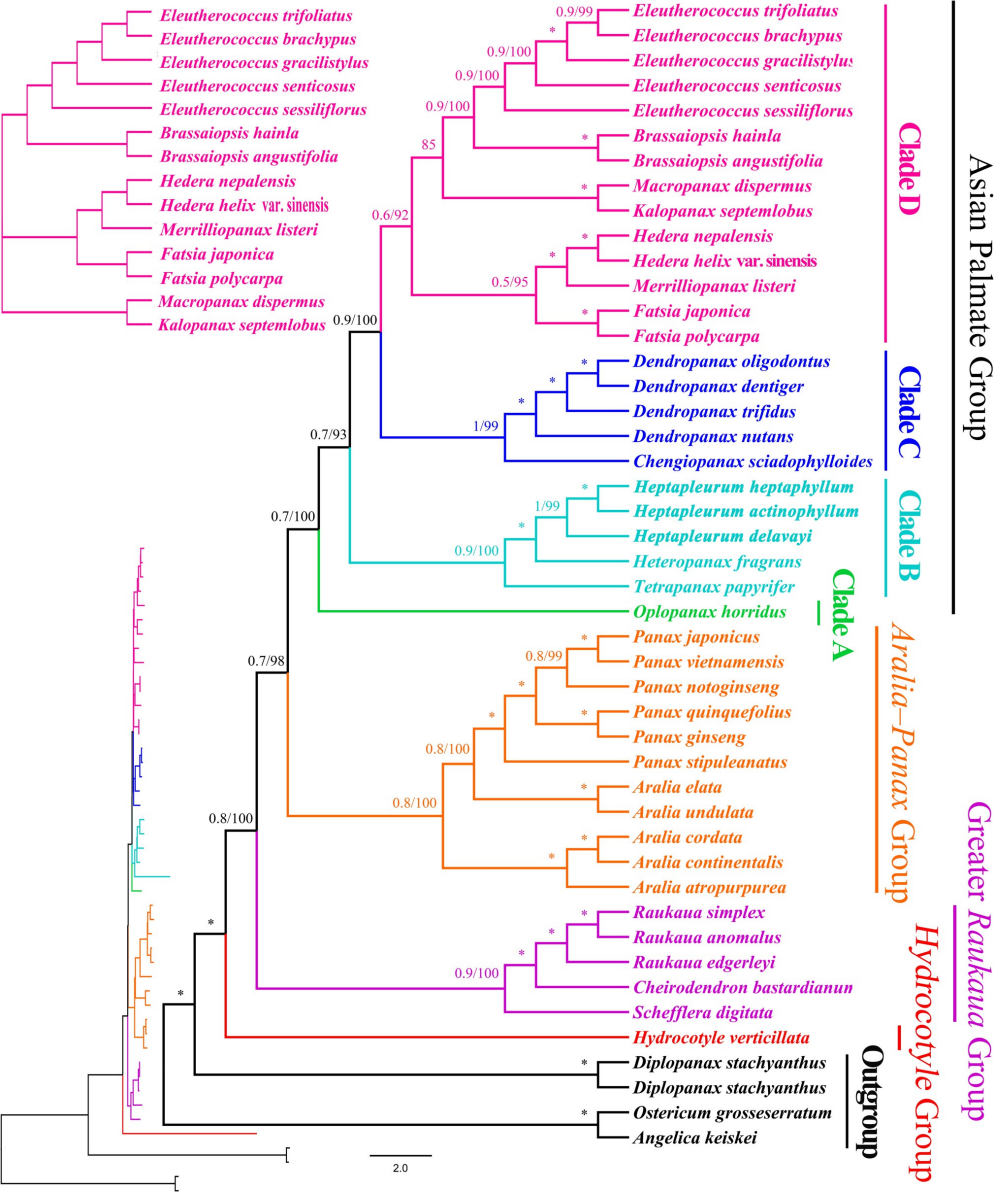
Molecular phylogenetic tree of 42 taxa of Araliaceae based on plastome sequences using unpartitioned Bayesian inference (BI) and maximum likelihood (ML). Numbers at each node are bootstrap support values. The tree is rooted in the plastome sequences of *Angelica keiskei*, *Ostericum grosseserratum*, and *Diplopanax stachyanthus*. Numbers associated with the branches are BI posterior probabilities (PP) and ML bootstrap value (BS), and asterisks (*) indicate BS/PP of 100/1.00.

In order to better understand the phylogenetic relationship among the 42 sequenced taxa from 19 genera of the Araliaceae, we have downloaded the corresponding sequences from GenBank (S1 Table). The BI and ML analysis of the complete plastid sequences of the main clades and most genera indicate that most phylogenetic relationships have a high degree of internal support (Fig 7). Araliaceae was divided into four groups: the *Hydrocotyle* group (BI-PP = 1.00, ML-BS = 100%), which included the *Hydrocotyle*. The Greater *Raukaua* Group (BI-PP = 0.9, ML-BS = 100%) included *Schefflera*, *Cheirodendron*, and *Raukaua*. The *Aralia*-*Panax* Group (BI-PP = 0.8, ML-BS = 98%) included *Aralia* and *Panax*. The Asian Palmate Group (BI‐PP = 0.7, ML-BS = 100%) can be divided into four small clades (A, B, C, and D). Clade A (BI‐PP = 0.7, ML-BS = 100%) included *Oplopanax*. Clade B (BI-PP = 0.9, ML-BS = 100%) included *Heptapleurum*, *Heteropanax*, and *Tetrapanax*. Clade C (BI‐PP = 1.00, ML-BS = 99%) included *Chengiopanax* plus *Dendropanax*. Clade D (BI-PP = 0.6, ML-BS = 92%) included *Fatsia*, *Hedera*, *Merrilliopanax*, *Brassaiopsis*, *Eleutherococcus*, *Kalopanax*, and *Macropanax*.

### Phylogenetic relationships among *Brassaiopsis* species

**Fig 8:**
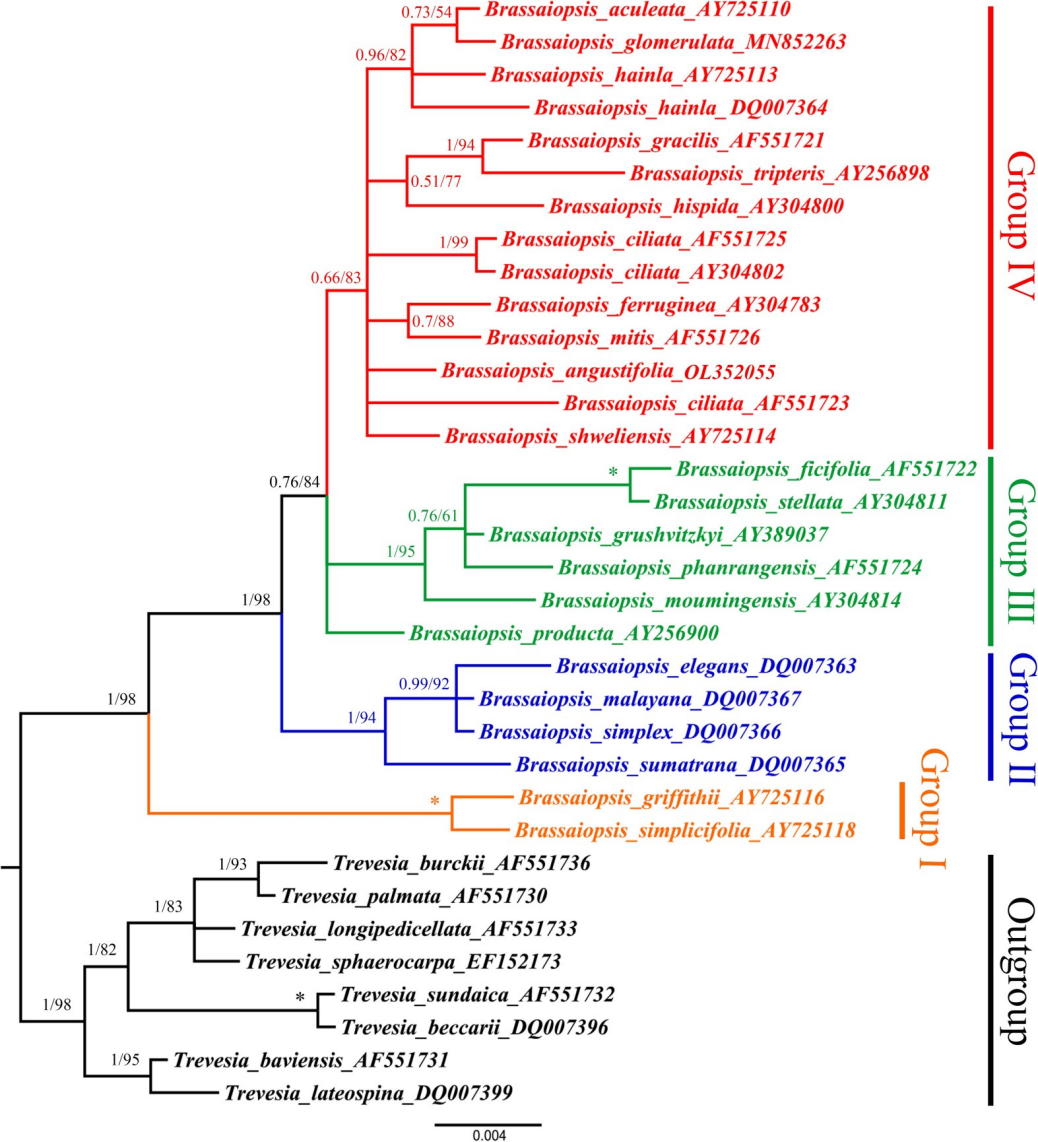
Bayesian inference (BI) and maximum likelihood (ML) strict tree illustrating the phylogeny of the genus *Brassaiopsis* based on nrDNA ITS datasets. Numbers associated with the branches are BI posterior probabilities (PP) and ML bootstrap values (BS), and asterisks (*) indicate BS /PP of 100/1.00.

To better understand the phylogenetic relationships among *Brassaiopsis* species the ITS sequences of 24 *Brassaiopsis* species were used in the evaluation, with eight *Trevesia* species as the outgroup. The phylogenetic tree divided the genus into four main groups (Fig 8). Group Ⅰ (BI-PP = 1.00, ML-BS = 100%) included *Brassaiopsis griffithii* C. B. Clarke and *Brassaiopsis simplicifolia* C. B. Clarke. Group Ⅱ (BI-PP = 1.00, ML-BS = 94%) included *Brassaiopsis elegans* Ridl., *Brassaiopsis malayana* J. Wen & Frodin, ined., *Brassaiopsis simplex* (King) B. C. Stone, and *Brassaiopsis sumatrana* Ridl.. Group Ⅲ (BI-PP = 1.00, ML-BS = 95%) included *Brassaiopsis ficifolia* Dunn, *Brassaiopsis grushvitzkyi* J. Wen, Lowry & T. H. Nguyên, *Brassaiopsis moumingensis* (Y. R. Ling) C. B. Shang, *Brassaiopsis phanrangensis* C. B. Shang, *Brassaiopsis producta* (Dunn) C. B. Shang, and *Brassaiopsis stellata* K. M. Feng. Group Ⅳ (BI-PP = 0.66, ML-BS = 83%) included *Brassaiopsis aculeata* Seem., *B*. *angustifolia*, *Brassaiopsis ciliata* Dunn, *Brassaiopsis ferruginea* (H. L. Li) G. Hoo, *Brassaiopsis glomerulata* (Blume) Regel, *Brassaiopsis gracilis* Hand.-Mazz., *B*. *hainla*, *Brassaiopsis hispida* Seem., *Brassaiopsis mitis* C. B. Clarke, *Brassaiopsis shweliensis* W. W. Sm., and *Brassaiopsis tripteris* (H. Lév.) Rehder.

## Discussion and conclusions

### Comparison of cp genomes in the Asian Palmate group species

This study revealed the entire cp genome of an endangered shrub, namely *B*. *angustifolia* in the family Araliaceae. The plastome with a length of 156,534 bp was larger than the published plastome of *B*. *hainla* [13], which both possess the typical angiosperm quadripartite structure (Fig 1). The cp genomes are highly conserved for mostland plants. The difference in the size of the IRs and intergenic spacers leads to differences between the cp genomes [42]. Reportedly, the *ycf1* and *ycf2* genes are located on the border between the IR region and the LSC and SSC regions, and there is incomplete replication of these two genes [13, 43]. Unlike *ycf2*, the lengths of the truncated *ycf1* genes were different among *B*. *angustifolia*, *B*. *hainla*, *E*. *brachypus*, *E*. *gracilistylus*, *E*. *senticosus*, *E*. *sessiliflorus*, *E*. *trifoliatus*, *K*. *septemlobus*, and *M*. *dispermus* (Fig 5). The gene *ycf1* passes through the SSC-IRb region, and the truncated *ycf1* gene was located in the LSC-IRa region. The change in length of the truncate *ycf1* gene directly caused the shrinkage of the IR region in the plastomes of *E*. *gracilistylus and K*. *septemlobus*. In addition, in the *M*. *dispermus*, there is a truncated *ycf1* gene in the LSC-IRa region; the same phenomenon exists in some species of other angiosperms [44]. Except for *B*. *angustifolia*, the *ndhF* gene is completely located in the SSC region in the other eight species, but the distance from the IRB/SSC boundary is different, which is consistent with the results obtained by Li et al. [13] in a study of seven species of Araliaceae.

### Analysis of mutational hotspots

Not all genetic mutation events are random, with some clustering as hotspots [45, 46]. These mutation dynamics created highly variable regions in the genome [43]. In *B*. *angustifolia* and *B*. *hainla* plastomes, we identified five highly variable loci, which included *psbⅠ*, *petN*-*psbM*-*trnD*-*GUC*, *trnT*-*GUU*-*psbD*, *ndhF*-*rpl32*, and *ycf1* (Fig 5A). In the Asian Palmate group, we identified five highly variable loci, which included *trnK*-*UUU*-*rps16*, *trnE*-*UUC*-*trnT*-*GGU*, *psbE*-*petL*, *ndhF*, and *ycf1* (Fig 5B). Compared with other highly variable regions, *ycf1* had the greatest genetic divergence among the nine sequenced plastid genomes of the Asian Palmate group (Fig 5B); from which, three highly variable loci were identified. These highly variable loci can be used for phylogenetic studies of the Araliaceae DNA barcode and at the species level. These results are partially congruent with those of Dong et al. [47] and Song et al. [48]. Therefore, Therefore, these associated regions can serve as barcodes as potential markers to reconstruct the phylogenetic relationship of Araliaceae.

The values of the nucleotide variability from the complete cp genomes among species of *B*. *angustifolia* and *B*. *hainla* were only 0.39%, and the nine species of the Asian Palmate group were 0.46%, which is similar to the nucleotide variability of two *Panax* L. species (0.40%) [49]; six *Persea* Mill., *Machilus* Nees, *Phoebe* Nees, and *Cinnamomum* Schaeff. species (0.32%) [50]; nine *Lindera* Thunb. species (0.48%) [51]; and 40 *Populus* L. species (0.36%) [52], which were much greater than the values of the two *Phoebe* Nees species (0.1%) [53], and three *Alseodaphne* Nees species (0.12%) [48], and were much less than the sequence divergence among the six *Cymbidium* Sw. species (3.70%) [54] and the five *Epimedium* L. species (3.97%) [55].

### Phylogenetic analysis of Araliaceae and *Brassaiopsis*

Previous molecular markers provided limited information to elucidate the relationship among Araliaceae plants. Many studies have attempted to resolve the relationships within Araliaceae using molecular markers of ITS or plastid-region data, but the relationship of Araliaceae was still unclear [1–3, 5, 7–9, 11]. In addition, studies show that increased sampling of taxa can greatly improve overall phylogenetic accuracy [56–58]. Our study and Valcárcel et al. [14] had found that using the whole plastomes, with an appropriate sampling, can greatly aid the relationship between the deep pedigrees of the Araliaceae. The family of Araliaceae, grouped in four main clades in molecular phylogenies (Greater *Rauakaua*, *Polyscias*–*Pseudopanax*, *Aralia*–*Panax*, and Asian Palmate) [3, 5, 10, 14]. From the results of our study, four branches of phylogenetic meaningful have been identified in deep lineages of the Araliaceae. The topological backbone of the phylogenetic genome obtained in this work agrees with those previously published [5, 10, 14]. Moreover, the problems of several major branches of the Araliaceae were solved. In the *Hydrocotyle* group, the genus of *Hydrocotyle* is located within Araliaceae and is a relatively primitive genus (Fig 7). In the Greater *Raukaua* Group, our phylogenetic analysis revealed a sister group containing three *Raukaua* species, one *Cheirodendron* species, and the *Schefflera digitata* species (Fig 7), with strong support, as in the previously published phylogenetic tree constructed with combinations of plastid markers of *trnL*-*trnF* [5], *trnD*-*trnT* plus *rpl16* [6], nuclear (ITS + external transcribed spacers [ETS]) plus plastid markers (*ndhF*-*rpl32*, *rpl32*-*trnL*, *trnK*-*rps16*, *trnH*-*psbA*) [7], and ITS plus plastid markers (*ndhF*, *trnL*–*F*, *rps16*, *atpB*–*rbcL*, *rpl16* and *psbA*–*trnH*) [10]. In the *Aralia*-*Panax* Group, our phylogenetic analysis revealed that *Aralia* is nested among the members of *Panax* (Fig 7), which is consistent with the cp data from Nicolas et al. [6], Valcárcel et al. [9], Mitchell et al. [10], Plunkett et al. [11] and Li et al. [13], the nuclear ribosomal DNA data from Wen et al. [3], and the nuclear plus plastid data from Li et al. [8]. In the Asian Palmate Group, *Oplopanax* that appears as sister to the remaining Asian Palmate group, as found in previous studies [7, 10, 14]. *Schefflera* has been shown to be polyphyletic and distributed in the main pedigree of the family [7–11]. With the deepening of the research, there has been a large taxonomic rearrangement of *Schefflera* that resulted in the description of five new or reinstated genera [7, 59–64]. The resurrection of *Heptapleurum* in an Asian clade formerly belonging to *Schefflera* (Araliaceae), and with the completion of these transfers, *Heptapleurum* is now the largest genus in Araliaceae [63, 64]. In the Clade B, our phylogenomic analysis shows that sisterhood contained three *Heptapleurum* species and *H*. *fragrans*, *Tetrapanax papyrifer* (Hook.) K. Koch and *Oplopanax horridus* (Smith.) Miq. (Fig 7), likewise significant support in the nuclear ribosomal ITS sequences [2, 11], ITS plus plastid markers [9, 10], and the cp genomes data [14]. Furthermore, these clade can be divided into four groups, with strong support, the backbones of the phylogenomic topologies obtained here are consistent with previously published phylogenetic relationships [6, 10, 11, 14].

The phylogeny of *Brassaiopsis* was estimated based on ITS sequences. In the deep lineages of the *Brassaiopsis* species, four branches of phylogenetic importance have been identified. In group Ⅰ, *B*. *griffithii* and *B*. *simplicifolia* were located in the earliest-diverging extant lineage within *Brassaiopsis* (Fig 8). Four species *B*. *elegans*, *B malayana*, *B*. *simplex*, and *B*. *sumatrana*, were located within group Ⅱ (Fig 8). In group Ⅲ, *B*. *ficifolia*, *B*. *grushvitzkyi*, *B*. *moumingensis*, *B*. *phanrangensis*, *B*. *producta*, and *B*. *stellata* formed a sister relationship. In the study of Mitchell et al. [1], *B*. *moumingensis* was found to be a sister with *B*. *grushvitzkyi*, and *B*. *stellata*. In group Ⅳ, *B*. *angustifolia* was a sister with the other 11 *Brassaiopsis* species (Fig 8). In addition, our phylogeny supports the following relationships: the sisterhood of the *B*. *hainla* and *B*. *aculeata*, flowed *B*. *glomerulata*, as found in previous studies [1, 2]. Because of the limited mutation sites in the ITS sequence, the systematic relationship among *Brassaiopsis* species has not been solved, and needs to be studied further.

## Supporting information

S1 Table Plant materials for data matrix Ⅰ. (DOCX)

S2 Table Plant materials for data matrix Ⅱ. (DOCX)

S3 Table Annotated genes of the *Brassaiopsis angustifolia* cp genome. (DOCX)
