# Supplementary material for: The complete plastid genome of the endangered shrub Brassaiopsis angustifolia (Araliaceae): Comparative genetic and phylogenetic analysis
Source: PLoS One. 2022 Jun 30;17(6):e0269819. doi: 10.1371/journal.pone.0269819 (PMC9246242; doi:10.1371/journal.pone.0269819)
Supplement: S1 Table — (DOCX) [file pone.0269819.s001.docx]

S1 Table: Plant materials for data matrix Ⅰ.

| Species | GenBank accession |
| --- | --- |
| *Chengiopanax sciadophylloides* | MK930365 |
| *Raukaua simplex* | MT385082 |
| *Raukaua edgerleyi* | MT385081 |
| *Raukaua anomalus* | MT385080 |
| *Cheirodendron bastardianum* | MT385071 |
| *Hydrocotyle verticillata* | HM596070 |
| *Panax notoginseng* | KJ566590 |
| *Panax quinquefolius* | KT028714 |
| *Panax japonicus* | KP036469 |
| *Panax vietnamensis* | KP036470 |
| *Panax ginseng* | NC006290 |
| *Panax stipuleanatus* | KX247147 |
| *Tetrapanax papyrifer* | MK943810 |
| *Heptapleurum heptaphyllum* | KT748629 |
| *Schefflera digitata* | MT385084 |
| *Heptapleurum actinophyllum* | MT385083 |
| *Heptapleurum delavayi* | KC456166 |
| *Oplopanax horridus* | MK943805 |
| *Merrilliopanax listeri* | MK943803 |
| *Macropanax dispermus* | MK943802 |
| *Kalopanax septemlobus* | KC456167 |
| *Hedera nepalensis var. sinensis* | MK130890 |
| *Hedera helix* | MK943800 |
| *Fatsia japonica* | KR021045 |
| *Fatsia polycarpa* | MK943798 |
| *Eleutherococcus trifoliatus* | MT754220 |
| *Eleutherococcus senticosus* | KY085901 |
| *Eleutherococcus sessiliflorus* | KT153019 |
| *Eleutherococcus gracilistylus* | KT153020 |
| *Eleutherococcus brachypus* | MN527993 |
| *Diplopanax stachyanthus* | KP318983 |
| *Diplopanax stachyanthus* | MG524991 |
| *Dendropanax oligodontus* | MT909827 |
| *Dendropanax dentiger* | KP271241 |
| *Dendropanax morbifer* | KR136270 |
| *Dendropanax nutans* | MK943797 |
| Species | GenBank accession |
| *Brassaiopsis hainla* | KC456164 |
| *Aralia atropurpurea* | MK778455 |
| *Aralia cordata* | MH778959 |
| *Aralia elata* | KT153023 |
| *Aralia undulata* | KC456163 |
| *Brassaiopsis angustifolia* | OK638200 |
| *Heteropanax fragrans* | MK943801 |
| *Ostericum grosseserratum* | KT852844 |
| *Aralia continentalis* | MG914654 |
| *Angelica keiskei* | MW125613 |
