# Supplementary material for: The complete plastid genome of the endangered shrub Brassaiopsis angustifolia (Araliaceae): Comparative genetic and phylogenetic analysis
Source: PLoS One. 2022 Jun 30;17(6):e0269819. doi: 10.1371/journal.pone.0269819 (PMC9246242; doi:10.1371/journal.pone.0269819)
Supplement: S2 Table — (DOCX) [file pone.0269819.s002.docx]

S2 Table: Plant materials for data matrix Ⅱ.

| Species | GenBank accession |
| --- | --- |
| *Brassaiopsis aculeata* (Buch.-Ham. ex D.Don) Seem. | AY725110 |
| *Brassaiopsis angustifolia* K.M.Feng | OL352055 |
| *Brassaiopsis ciliata* Dunn | AF551723 |
| *Brassaiopsis elegans* Ridl. | DQ007363 |
| *Brassaiopsis ciliata* Harms | AF551725 |
| *Brassaiopsis ferruginea* (H.L.Li) G.Hoo | AY304783 |
| *Brassaiopsis ficifolia* Dunn | AF551722 |
| *Brassaiopsis glomerulata* (Blume) Regel | MN852263 |
| *Brassaiopsis gracilis* Hand.-Mazz. | AF551721 |
| *Brassaiopsis griffithii* C.B.Clarke | AY725116 |
| *Brassaiopsis grushvitzkyi* J.Wen, Lowry & T.H.Nguyên | AY389037 |
| *Brassaiopsis hainla* (Buch.-Ham.) Seem. | AY725113 |
| *Brassaiopsis hispida* Seem. | AY304800 |
| *Brassaiopsis malayana* J. Wen & Frodin, ined. | DQ007367 |
| *Brassaiopsis mitis* C.B.Clarke | AF551726 |
| *Brassaiopsis moumingensis* (Y.R.Ling) C.B.Shang | AY304814 |
| *Brassaiopsis ciliata* Forrest ex W.W.Sm. | AY304802 |
| *Brassaiopsis phanrangensis* C.B.Shang | AF551724 |
| *Brassaiopsis hainla* (Wall.) R.N.Banerjee | DQ007364 |
| *Brassaiopsis producta* (Dunn) C.B.Shang | AY256900 |
| *Brassaiopsis shweliensis* W.W.Sm. | AY725114 |
| *Brassaiopsis simplex* (King) B.C.Stone | DQ007366 |
| *Brassaiopsis simplicifolia* C. B. Clarke | AY725118 |
| *Brassaiopsis stellata* K.M.Feng | AY304811 |
| *Brassaiopsis sumatrana* (Miq.) Ridl. | DQ007365 |
| *Brassaiopsis tripteris* (H.Lév.) Rehder | AY256898 |
| *Trevesia baviensis* | AF551731 |
| *Trevesia beccarii* Boerl. | DQ007396 |
| *Trevesia burckii* Boerl. | AF551736 |
| *Trevesia lateospina* Jebb | DQ007399 |
| *Trevesia longipedicellata* Grushv. & Skvortsova | AF551733 |
| *Trevesia palmata* (Roxb. ex Lindl.) Vis. | AF551730 |
| *Trevesia sphaerocarpa* Grushv. & Skvortsova | EF152173 |
| *Trevesia sundaica* Miq. | AF551732 |
