# Supplementary material for: The complete plastid genome of the endangered shrub Brassaiopsis angustifolia (Araliaceae): Comparative genetic and phylogenetic analysis
Source: PLoS One. 2022 Jun 30;17(6):e0269819. doi: 10.1371/journal.pone.0269819 (PMC9246242; doi:10.1371/journal.pone.0269819)
Supplement: S3 Table — (DOCX) [file pone.0269819.s003.docx]

S3 Table: Annotated genes of the *Brassaiopsis angustifolia* cp genome.

| Group of gene | Name of gene |
| --- | --- |
| **Photosynthesis-related genes** |  |
| Rubisco | *rbcL* |
| Photosystem | *IpsaA*, *psaB*, *psaC*, *psaI*, *psaJ* |
| Assembly and stability of photosystem I | ***ycf3*, *ycf4* |
| Photosystem II | *psbA*, *psbB*, *psbC*, *psbD*, *psbE*, *psbF*, *psbH*, *psbI*, *ppsbJ*, *psbK*, *psbL*, *psbN*, *psbT*, *psbZ* |
| ATP synthase | *atpA, atpB*, *atpE*, **atpF*, *atpH*, *atpI* |
| Cytochrome b/f complex | *petA*, **petB*, **petD*, *petG*, *petL*, *petN* |
| Cytochrome c synthesis | *ccsA* |
| NADPH dehydrogenase | **ndhA*, **ndhB*^2^, *ndhC*, *ndhD*, *ndhE*, *ndhF*, *ndhG*, *ndhH*, *ndhI*, *ndhJ*, *ndhK* |
| **Transcription- and translation-related genes** |  |
| Transcription | *rpoA*, *rpoB*, **rpoC1*, *rpoC2* |
| Ribosomal proteins | *rps2*, *rps3*, *rps4*, *rps7*^2^, *rps8*, *rps11*, **rps12*, *rps14*, *rps15*, **rps16*, *rps18*, *rps19*, ***rpl2*^2^, *rpl14*, **rpl16*, *rpl20*, *rpl22*, *rpl23*^2^, *rpl32*, *rpl33*, *rpl36* |
| Translation initiation factor | *infA* |
| **RNA genes** |  |
| Ribosomal RNA | *rrn5*^2^, *rrn4.5*^2^, *rrn16*^2^, *rrn23*^2^ |
| Transfer RNA | **trnA–UGC*^2^, *trnC–GCA*, *trnD–GUC*, *trnE–UUC*, *trnF–GAA*, *trnG–GCC*, **trnG–UCC*, *trnH–GUG*, *trnI–CAU*^2^, **trnI–GAU*^2^, **trnK–UUU*, *trnL–CAA*^2^, **trnL–UAA*, *trnL–UAG*, *trnfM–CAU*, *trnM–CAU*, *trnN–GUU*^2^, *trnP–UGG*, *trnQ–UUG*, *trnR–ACG*^2^, *trnR–UCU*, *trnS–GCU*, *trnS–GGA*, *trnS–UGA*, *trnT–GGU*, *trnT–UGU*, *trnV–GAC*^2^, **trnV–UAC*, *trnW–CCA*, *trnY-GUA* |
| **Other genes** |  |
| RNA processing | *matK* |
| Carbon metabolism | *cemA* |
| Fatty acid synthesis | *accD* |
| Proteolysis | ***clpP* |
| **Genes of unknown function** |  |
| Conserved reading frames | *ycf1*, *ycf2*^2^ |
| **Pseudogenes** |  |
| — | *ycf15*^2^ |

Note: A single asterisk (*) preceding gene names indicate intron-containing genes, double asterisks (**) preceding gene names indicate two introns in the gene, and (2) indicates that the gene is located in the IR region.
